# Routine genomic surveillance in a military healthcare facility detected a community-based Group A *Streptococcus* outbreak associated with grappling sports

**DOI:** 10.1017/ice.2025.10367

**Published:** 2026-02

**Authors:** Germán G. Vargas-Cuebas, William Stribling, Melissa J. Martin, Shannon Gettings, Rhonda Wells, Maureen Sevilla, Kathryn Polaskey, Lan Preston, Yoon I. Kwak, Patrick T. Mc Gann, Francois Lebreton, Jason W. Bennett

**Affiliations:** 1 Multidrug-Resistant Organism Repository and Surveillance Network (MRSN), Diagnostics and Countermeasures Branch, Center for Infectious Disease Research, Walter Reed Army Institute of Research, Silver Spring, MD, USA; 2 Public Health Clinic, Department of Public Health, Womack Army Medical Center, Fort Bragg, NC, USA

## Abstract

An outbreak of *emm92*/ST82 *Streptococcus pyogenes* was detected through prospective genomic surveillance at a military treatment facility. Twenty-one of twenty-six patients had confirmed epidemiological links to grappling sports. One case resulted from household transmission. The benefits of routine surveillance extend beyond the hospital environment enabling the detection of community-driven transmission.

## Introduction

*Streptococcus pyogenes*, also known as group A *Streptococcus* (GAS), is an opportunistic pathogen capable of asymptomatic colonization of skin and mucosa or causing diseases ranging from cellulitis to life-threatening invasive infections (iGAS).^1^ Beyond its global health burden, GAS have been linked to outbreaks and substantial morbidity and mortality among military personnel, partly due to physical training and living in close quarters.^2^ A United States (U.S.) military review documented 17 GAS outbreaks involving thousands of cases over a single decade, highlighting the need for effective surveillance.^2^

GAS type *emm92,* as defined by molecular typing of the M protein, is among the most prevalent U.S. *emm* types causing iGAS, primarily in community-associated infections.^3,4^ Here, we describe a GAS *emm92/*ST82 outbreak detected by genomic surveillance at a military treatment facility (MTF) by the Multidrug-Resistant Organism Repository and Surveillance Network (MRSN). Genomic and epidemiological analyses revealed high clonality among isolates and a link to grappling-sports training at multiple gyms surrounding a U.S. military base.

## Methods

### Bacterial isolates, whole genome sequencing (WGS), and bioinformatics analysis

GAS isolated colonies were identified in the MTF microbiology laboratory using a Bruker (MALDI-TOF) instrument and sent to the MRSN for molecular analysis. DNA extraction and library preparation followed published protocols.^5^ Whole-genome sequencing was performed on Illumina MiSeq or NextSeq-2000 instruments (2 × 300 bp or 2 × 150 bp). Reads were trimmed with Bbduk v38.96, classified and screened for contamination using Kraken2, and assembled *de novo* with Shovill v1.0.9 (≥200 bp contigs, ≥50x coverage).

Genome annotation and *in silico* typing used AMRFinderPlus v3.12.8 for AMR gene detection, emmtyper v0.1.0 for *emm* typing, and mlst v2.22 for sequence typing (ST). Core-genome MLST (cgMLST) was conducted in SeqSphere+ v7.7.2 using the *S. pyogenes* scheme applying a < 5 allele threshold to define potential clusters and was visualized in iTOL.^6,7^ SNP analysis was performed with Snippy v4.4.5 (earliest isolate as reference) and Pilon v1.23 for error correction.

### Epidemiological analysis and infection and prevention control (IPC) response

Epidemiological investigation combined patient interviews and retrospective chart review to identify potential exposures and links among confirmed cases. Data were used to assess associations with grappling sports, training sites, and household contacts. Intervals between positive culture and interview ranged from 27 to 36 days for 2025 cases, and could extend to 384 days for initial cases. Infection prevention efforts, led by the Department of Public Health (DPH), included community outreach, environmental inspections of gyms, and staff education on hygiene and disinfection. Rapid genomic data supported timely interventions and refinement of control strategies. While recall bias may affect the accuracy of individual risk factor assessment, consistent associations with grappling sports indicated an overall epidemiological trend.

## Results

**Figure 1.**
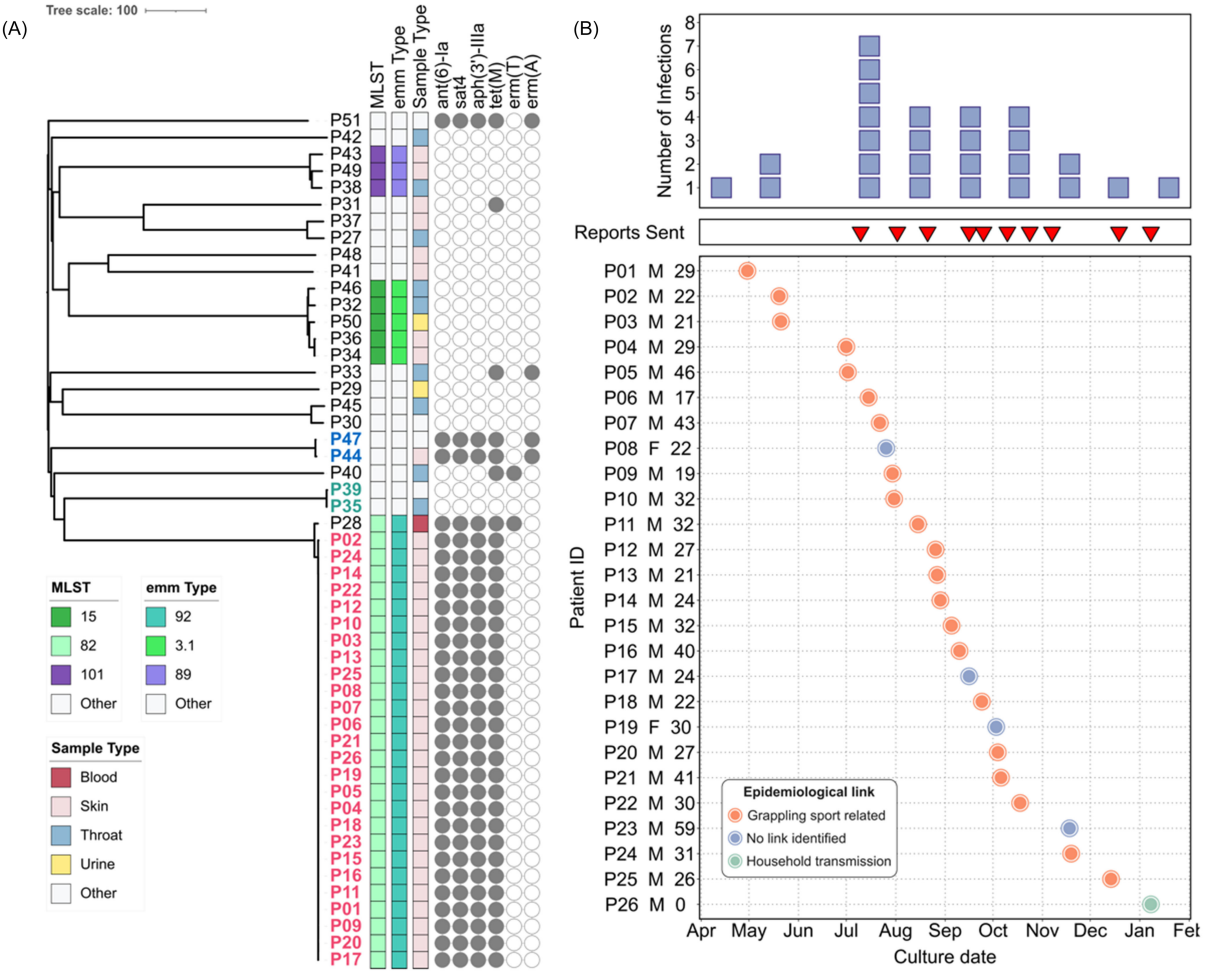
ST-82 outbreak *S. pyogenes* (GAS) linked to grappling-based sports. (A) Neighbor-joining tree based on cgMLST of 51 GAS isolates from one MTF (March 2024 –February 2025). (B) Number of ST-82 outbreak infections over 9 months (top panel), alert reports sent (middle panel), and patient chart (bottom panel). Orange, grappling sport related; green, suspected household transmission.

Starting in 2024, because of its relevance to military medicine, the MRSN expanded its genomic surveillance program^5^ to include GAS. Consequently, all GAS isolates cultured from all types of clinical specimens from both inpatients and outpatients were collected for genomic analyses. Between March 2024 and February 2025, 51 GAS isolates representing 15 distinct STs were recovered from 51 patients at a single MTF (Table S1). ST-82 (*n* = 27), ST-15 (*n* = 5), and ST-101 (*n* = 3) were the most represented. Retrospective cgMLST analysis revealed three clusters of high genetic relatedness (Figure 1A), two of which involved two isolates from two outpatients each: P35 and P39 (ST-28, 1 SNP apart); and P44 and P47 (ST-433 isolates, 2 SNPs apart). At the time of detection, reports were sent to the hospital initiating infection prevention and control (IPC) investigations that, in the absence of patient/staff overlap, ruled against a likely nosocomial transmission. The third cluster grouped isolates collected from 26 outpatients and is hereby described as the ST-82 outbreak (Table 1).

**Table 1.** Metadata of patients associated with *S. pyogenes* (GAS) ST-82 outbreak

Isolate	Patient	Days since initial outbreak case	Sex	Age	Grappling-sports Training	Symptoms
136128	P1	0	M	29	Yes	Erythema, abscess, purulent drainage.
137296	P2	20	M	22	Yes	Erythema, abscess, purulent drainage.
137298	P3	21	M	21	Yes	Lymphadenopathy
140752	P4	62	M	29	Yes	Edema, abscess, purulent drainage.
140754	P5	63	M	46	Yes	Boil or furuncle.
141740	P6	76	M	17	Yes	Dermatitis.
142545	P7	83	M	43	Yes	Abscess, purulent drainage.
142550	P8	87	F	22	No	Dermatitis.
144444	P9	91	M	19	Yes	Abscess, pain.
144445	P10	92	M	32	Yes	Purulent drainage.
143697	P11	107	M	32	Yes	Dermatitis.
144670	P12	118	M	27	Yes	Chronic wound.
144672	P13	119	M	21	Yes	Erythema, purulent drainage.
144676	P14	121	M	24	Yes	Purulent drainage.
144933	P15	128	M	23	Yes	Boil or furuncle.
145177	P16	133	M	40	Yes	Chronic wound.
146326	P17	139	M	24	No	Dermatitis, abscess.
146462	P18	147	M	22	Yes	Abscess.
146738	P19	156	F	30	No	Boil or furuncle.
146740	P20	157	M	27	Yes	Purulent drainage.
147239	P21	159	M	41	Yes	Dermatitis, abscess, pustular drainage.
147383	P22	171	M	30	Yes	Abscess, erythema, purulent drainage.
150348	P23	202	M	59	No	Abscess.
150350	P24	203	M	31	Yes	Erythema.
151853	P25	228	M	26	Yes	Erythema.
152788	P26	253	M	0	No*	Boil or furuncle.

* Suspected household transmission event.

All 26 ST-82 outbreak isolates belonged to *emm* type 92/ST-82 and carried the resistance determinants *ant*(6) and *aph*(3’)-III, *sat4*, and *tetM*, conferring resistance to aminoglycoside, streptothricin, and tetracycline, respectively (Figure 1A). Variant analysis confirmed the genetic relatedness between these isolates (0–2 SNPs). An additional *emm* type 92/ST-82 isolate (outpatient P28) was confirmed by SNP analysis to be a distinct strain (31 SNPs apart).

Because sequencing and genomic analysis were performed prospectively, multiple alerts were sent to the IPC staff as new cases were detected. In July 2024, the initial ST-82 outbreak cluster involving 3 patients (P1–P3) was identified (Figure 1B). Subsequent reports describing 23 additional cases (P4–P26) were sent to the MTF, with the base DPH notified in September 2024. The base DPH epidemiological analysis revealed that 21 cases (81%) had direct epidemiological links to grappling sports (ie Army Combatives training, jiu jitsu, or wrestling). One case involving an infant was linked to household transmission likely from a sibling who participated in jiu jitsu at one of the previously identified facilities (Figure 1B). Cases spanned nine different training facilities, four grappling-focused, with eleven cases associated with a single facility (Table S2). Environmental swabs (*n* = 19) of mats and equipment at this facility were negative for GAS. When compared with the rest of GAS cases within the same MTF and period, the 26 outbreak-associated patients displayed a significantly higher male to female ratio (M:F ratios = 12 vs. 1.8, respectively; Fisher’s exact test *p*-value = 0.0207), while median ages were comparable (ST-82 outbreak cases median age: 27 years, IQR: 9.5; other GAS cases median age: 28 years; IQR: 10) (Table S1 and Figure. 1). No additional epidemiological links were identified.

## Discussion

We report a GAS community outbreak linked to grappling sports. Twenty-six outpatients over 9 months presented with skin and soft tissue infection, a condition common in grappling sports involving skin-to-skin contact and abrasions.^8^ Individuals were largely male, likely reflecting the bias in populations performing combative sports and active-duty service members.^9,10^ Twenty-one individuals had direct links to training facilities, and one infant patient was indirectly related through a family member; however, the suspected household transmission remains unconfirmed due to lack of samples from subjects involved.

All 26 ST-82 isolates were nearly identical (0–2 SNPs) despite been associated with multiple training facilities (42% form a single facility). Swabbing of high-contact surfaces at this facility ruled out an environmental reservoir, suggesting skin-to-skin transmission during combat as the most likely route of transmission. Participation in regional competitions (reported during interviews) may have facilitated the spread across multiple training facilities, but further investigations are needed for corroboration. The association of this outbreak with nine grappling training facilities underscores the need for proactive prevention measures, including routine skin assessments, strict enforcement of hygiene protocols, regular disinfection of equipment and mats, and educational efforts.

Although epidemiological investigations revealed community-driven transmission, detection relied on routine genomic surveillance at the MTF by the MRSN. Non-DoD beneficiaries or mildly affected individuals who did not seek medical care were likely missed, indicating this report likely underestimates the true case count linked to this outbreak. Surveillance at this location continues with six additional closely related cases (1–2 SNPs apart from outbreak isolates) detected between April and August 2025. Four of these patients participated in grappling-sports training, reinforcing the link between grappling-based activities and this GAS outbreak.

## Supporting information

Vargas-Cuebas et al. supplementary material 1 Vargas-Cuebas et al. supplementary material

Vargas-Cuebas et al. supplementary material 2 Vargas-Cuebas et al. supplementary material

## Data Availability

Genomes described herein have been deposited at GenBank under BioProject PRJNA1257432.

## Supplementary material

The supplementary material for this article can be found at https://doi.org/10.1017/ice.2025.10367.
